# On the Better Performance of Pianists with Motor Imagery-Based Brain-Computer Interface Systems

**DOI:** 10.3390/s20164452

**Published:** 2020-08-10

**Authors:** José-Vicente Riquelme-Ros, Germán Rodríguez-Bermúdez, Ignacio Rodríguez-Rodríguez, José-Víctor Rodríguez, José-María Molina-García-Pardo

**Affiliations:** 1 Consejería de Educación y Cultura de la Región de Murcia, E30003 Murcia, Spain; josevicente.riquelme@murciaeduca.es; 2 University Center of Defense, San Javier Air Force Base, Ministerio de Defensa-Universidad Politécnica de Cartagena, E30720 Santiago de la Ribera, Spain; german.rodriguez@cud.upct.es; 3 Departamento de Ingeniería de Comunicaciones, ATIC Research Group, Universidad de Málaga, E29071 Málaga, Spain; ignacio.rodriguez@ic.uma.es; 4 Departamento de Tecnologías de la Información y las Comunicaciones, Universidad Politécnica de Cartagena, E30202 Cartagena, Spain; josemaria.molina@upct.es

**Keywords:** brain-computer interface, motor imagery, machine learning, internet of things, pianists

## Abstract

Motor imagery (MI)-based brain-computer interface (BCI) systems detect electrical brain activity patterns through electroencephalogram (EEG) signals to forecast user intention while performing movement imagination tasks. As the microscopic details of individuals’ brains are directly shaped by their rich experiences, musicians can develop certain neurological characteristics, such as improved brain plasticity, following extensive musical training. Specifically, the advanced bimanual motor coordination that pianists exhibit means that they may interact more effectively with BCI systems than their non-musically trained counterparts; this could lead to personalized BCI strategies according to the users’ previously detected skills. This work assessed the performance of pianists as they interacted with an MI-based BCI system and compared it with that of a control group. The Common Spatial Patterns (CSP) and Linear Discriminant Analysis (LDA) machine learning algorithms were applied to the EEG signals for feature extraction and classification, respectively. The results revealed that the pianists achieved a higher level of BCI control by means of MI during the final trial (74.69%) compared to the control group (63.13%). The outcome indicates that musical training could enhance the performance of individuals using BCI systems.

## 1. Introduction

A brain-computer interface (BCI) system uses various techniques to recognize brain activity and transform this biological signal into a command that can be used by computer systems to complete certain tasks [1]. At the same time, it provides feedback to the user on how the intentions are being transformed into actions. In short, a BCI system transforms mental activity into a command that can affect the surroundings without the user making a physical effort. This command can be used for various applications, such as moving orthopedic prostheses through imagery [2]. Using a similar strategy, a recent work explored the use of noninvasive neuroimaging to enhance the control of a robotic device to complete daily tasks [3]. Similarly, BCIs are also used to synthesize speech by people who are unable to communicate due to neurological impairments [4]. BCIs have also been used to improve rehabilitation after a stroke, translating brain signals into the intended movements of a paralyzed limb [5] and, more recently, for the control through thought of applications for smart homes or robots within the Internet of Things (IoT) context [6,7].

It has been established that musical practice involves the activation of numerous areas of the brain that carry out different yet complementary functionalities to perform the complex task of playing a musical instrument, including reading a score, performing complex, highly specific movements, performing from memory, increased attention and concentration levels during performance, controlling the tuning of the instrument, and even improvisation. It is not unreasonable to conclude that musicians’ brains have anatomical and functional differences compared to non-practitioners [8]. Therefore, the musician’s brain offers a unique example with which to investigate what influence musical exercise can have on brain structures.

Of all the abilities musicians possess, their high motor coordination capacity is especially interesting. Engaging in a musical profession requires years of practice, with a great deal of time each day devoted to rehearsing specific and concrete movements involving the fine musculature of the hands or body. This undoubtedly effects a greater skill in handling the instrument, which is reflected in the individual’s brain structures. The hand movements eventually display greater precision and coordination after the years of practice necessary to become a good musician.

Motor imagery, as a strategy to control a BCI system, has been investigated in groups of people with different characteristics, such as airplane pilots carrying out motor imagery tasks [9]. The present work aims to explore the handling of a BCI system through motor imagery by musicians, specifically pianists, for potential applications. It is hereby assumed that motor imagery control would be more intuitive for pianists and they would, therefore, achieve better performance, building on their previous muscle memory experience. Hence, this research aims to examine motor imagery in pianists to instantiate the benefits of musical education as well as show whether it is possible to consider a personalized BCI strategy for each subject according to their previously identified skills.

Following these introductory aspects, Section 2 outlines the prior work on the subject. Section 3 explains the experimental phase, detailing the characteristics of the test subjects, the resources used, and the method of data collection. The results are presented in Section 4. The work ends with the conclusions and future avenues for research in Section 5.

## 2. Music Training and Motor Imagery

Musicians’ brains have been studied extensively over the last few years as a textbook case of neuroscience research. Given that the main theme of this paper is to explore whether musical training (specifically in pianists) has an influence on the control of a BCI system through motor imagery, this section will present the previous work supporting this hypothesis.

Numerous examples in the literature have analyzed the neuroscientific foundations of music. The work of [10] presents an exhaustive review of how musical production and perception influence cognitive abilities, involving the areas of the auditory cortex and the motor cortex. Münte et al. [8] already examined the neuroanatomical peculiarities of musicians’ brains, highlighting their greater neuroplasticity. Later works, such as [11], delved further into the preceding ideas. Indeed, learning to play an instrument is a highly complex task that involves the interaction of higher-order cognitive functions and leads to behavioral, structural, and functional changes in the brain. Consequently, due to the need to constantly engage in musical practice, multiple differences have been shown to appear in the following areas of musicians’ brains:

- Corpus callosum. This connects both cerebral hemispheres. It has been observed to be larger in professional musicians, especially those who began musical studies at an early age [12]. This larger size implies a higher interhemispheric transfer rate.
- Motor cortical regions. Numerous studies have shown that professional musicians have a much greater symmetry between the two hemispheres and that the representation of the hand in the motor cortex is much larger in musicians [13].
- Cerebellum. This structure, among other cognitive functions, is involved in the temporal sequencing and coordination of movements, which is undoubtedly fundamental in musical praxis. It has been demonstrated that musicians possess a greater cerebellum volume [14]. Furthermore, it has been proven that this greater size is related to the intensity of musical training (number of hours practiced per day throughout life) as well as the fact of having initiated this training at an early age.
- Brain stem. This structure deals with basic sensory mechanisms. It has been possible to register faster reactions in musicians responding to certain musical and linguistic stimuli [15].

Taking the above into account, it can be stated that there is a positive correlation between the intensity and frequency of musical practice and the anatomical changes in the brain structures.

Musical performance is the complex activity responsible for these structural changes. It is considered extremely complex as it requires three special skills, i.e., the basic motor controls of coordination, sequencing, and spatial organization of movement [16]. Coordination refers to a good arrangement of the rhythmic aspect of music, while sequencing and spatial organization of movement imply the musician playing the notes on the instrument. It has been observed that more complex note sequences require the activity of structures such as the basal ganglia, dorsal premotor cortex, and cerebellum. The spatial organization of the different movements required to play an instrument involves the integration of different channels of spatial, sensory, and motor information, whereby the activation of the parietal, sensorimotor, and premotor cortex is observed.

In addition, audiomotor interactions occur in the brain when performing music as well as in the passive activity of listening to music. The premotor cortex is the link between the auditory system and the motor control, and for this activation to occur, the person must have an identified sound/action relationship [16].

It is evident that musical practice involves an increase in motor skills. In concrete studies focused on secondary motor areas, musicians show a much smaller activation area in these zones than non-musicians, demonstrating that pianists require smaller neural networks than non-musicians when it comes to motor skills, which in turn indicates that they are more efficient in controlling movements [17,18]. For example, practicing a complex fingering task for several months leads to an increase of approximately 25% in the primary motor area activation (M1). Furthermore, while musicians repeating the same sequence show a small area of activation (habituation) when a new music piece is trained for the first time, there is a larger area of activation (enhancement) [19]. During a fingering task performed by musicians and non-musicians, the former showed a rapid increase in the primary motor cortex (M1), while this was not seen in the latter [20].

Another study has shown that there are functional changes in the brains of children after 15 months of music training [10]. Two groups were studied, one receiving musical training and the other not. In the initial phase, the authors of such study found no differences between the groups. However, after the indicated period, it was found that the children who had been trained improved in motor control and melodic-rhythmic tasks, which supports the fact that the changes seen in adults (musicians) are due to musical practice.

As can be seen, intensive musical training leads not only to structural but also functional modifications in the youth’s brain. However, these changes can also be induced in adults, thus preserving areas of gray and white substance [21].

With this, functional changes in the motor cortex occur. The motor cortex changes when performing simple piano exercises with five fingers and also increases the activity of the basal ganglia and the cerebellum. Most significantly, these changes take place either if the practice is performed physically or mentally [15]. In fact, the musician’s brain is a paradigm of neuroplasticity [8].

Considering the above, it seems clear that musicians have greater motor coordination capacity than non-musicians, derived from intensive musical practice. However, the present study focuses on the performance of motor imagery. The question is whether undertaking music training in a mental (imagined) way can improve both motor coordination and the actual exercise. Indeed, using transcranial magnetic stimulation (TMS), Pascual-Leone et al. [22] demonstrated that the mere mental practice of an exercise in fingering a specific sequence for two hours a day over five days was sufficient to produce a certain reorganization of the motor cortex.

Subsequently, it was shown that the areas involved in motor imagery are approximately the same as those activated in real musical perception [23]. The same conclusion was drawn in another study [24] examining the MRI activity of seven pianists and seven participants with no musical experience. A few years earlier, [25] had indicated that many professional athletes and musicians can use movement imagery to improve their motor skills.

In the specific case of pianists, the most recent study by Zabielska-Mendyk et al. [26] compared the EEG patterns of pianists and non-pianists while executing both real and imagined fingering of different complexities. The power of the alpha and beta bands (mu rhythm modulation) decreased with decreasing fingering complexity (in both real and imagined cases), and this only occurred with the pianists; the non-musicians did not exhibit this attenuation. This capacity varies according to the experience in years that the musician accumulates, which is acquired progressively, as per [27]. This result already suggests a different behavior in terms of BCI performance.

Throughout these reviewed studies, it seems clear that circumstances may exist that improve the motor imagery skills of some individuals over those of others. However, how this advantage can lead to better performance when using a BCI system has thus far not been described. The presence of significant differences in this performance was investigated by Dobrea et al. [28], while it has been suggested that certain individual traits act as precursors in predicting performance in using a BCI system [29]; other predictors include spatial (motor) skills, which encompass the practice of a musical instrument.

Notably, some people appear to have no capacity to control a BCI, a phenomenon that numerous analysts in the literature have termed “BCI illiteracy” [30]. Hereby, it should be noted that BCIs are generally not easy to control, and even with proper instruction, some users cannot control their systems as desired. Nevertheless, BCI illiteracy is an inadequate concept for clarifying the trouble that users can have when working with BCI frameworks. First, it is a methodologically frail idea that depends on the imperfect assumption that BCI users have physiological or useful qualities that forestall capable performance during BCI use [31]. Second, the term BCI illiteracy invites a comparison between learning to use BCIs and spoken or written language acquisition. Hence, to avoid conceptual snares in terms of how BCI use may or may not relate to language learning, some researchers have chosen to use the term “BCI inefficiency”.

Various aspects associated with music are widely used to control BCI systems. For example, Makeig et al. [32] set out to control a BCI system by recreating the emotions produced by different pieces of music. In other words, they sought to recognize the emotions generated by a melody. Up to 84% success in certain experiments was achieved with this method.

Next, as this article uses motor imagery to control a BCI system, it reviews the literature evaluating the performance achieved through this control strategy. In [30], the authors explored the different outcomes in motor imagery achieved with different participants, drawing a distinction between the variability among different participants and that among different states of the same participant. They covered previous works, comparing personal characteristics, psychological mood, and anatomical and physiological aspects, concluding that all these components are essential when discussing future BCI performance.

Randolph et al. [33] developed what has become one of the main works through which we delimit our study area and define our research hypothesis. The authors considered factors such as age, sex, playing sports, playing video games, taking psychiatric medication, and playing a musical instrument. They concluded that a series of personal characteristics influence the modulation of the mu rhythm, leading to better outcomes in the control of a BCI. Specifically, having motor dexterity of the hands leads to better control over a BCI device. Furthermore, they examined the characteristics of age, time spent typing per day, the performance of hand-arm movements, and whole-body movements. They found that both age and hand-arm movements correlate positively with the ability to modulate rhythm induced by both real and imagined movements. The possibility of doing sports or playing a musical instrument is implied within these movements.

A large proportion of motor training occurs when the brain anticipates a movement being executed, i.e., if substantial repetition occurs prior to a movement, cerebral training results in a wave anticipating the movement [34]. Overall, some previous studies have pointed to the importance of the chosen movement used to control the BCI system. For instance, [28] discussed different types of tasks, e.g., motor, mathematics, and linguistics skills, whereby the motor tasks included movements of the fingers of the hand (left/right) and arm (left/right), while Soriano et al. [35] reviewed the different imagined movements that have been used in BCI. In terms of specific results, [36] showed that the movements of the right hand generate a differentiated signal on the EEG and cause hemodynamic activity in the motor cortex of the left hemisphere, detected by fMRI. When such movements are imagined, they generate similar, but less stable, patterns [37].

Other movements that have been examined in this context include grasping with the hand [38], the general use of the fingers (fingering) [39], the use of the index finger [40], the use of the big toe [41], and the maximum contraction of the hand [37]. Soriano et al. [35] undertook a comparison of these movements. However, despite the wealth of previous research, there is no specific study analyzing the performance of BCI system control by professional pianists using their high-level skills with fingers and hands.

## 3. Methodology

### 3.1. Sample Characteristics

This work aims to investigate whether pianists can control a BCI system by means of motor imagination more efficiently than non-musicians. To this end, we conducted an experiment analyzing the BCI performances of a group of pianists and a group of non-pianists (generally non-musicians), which functioned as a control group. The characteristics of the participants in both groups were collected, such as their sex and age as well as other more specific features, such as musical practice, the practice of other activities that involve finger movement (typing, video games), and playing sports.

The sample size in this experiment was 8 individuals, with 4 in each group. During the experiment, all participants were duly informed of how it would be conducted (passive and non-invasive measurement) as well as how the collected data would be handled. Furthermore, the anonymity of their personal and EEG data was guaranteed at all times. All experimentation was conducted in accordance with the Declaration of Helsinki and the ethics committees of the involved institutions were asked for approval before the sessions began.

In the case of the pianist group, participants were sought who had at least 10 years of musical training. Both men and women were included, some of whom were still in the process of musical training. The group rehearsed for an average of 5.75 h a day, ensuring significant skill in motor coordination. The characteristics of this group are summarized in Table 1.

Various factors were considered in the creation of the control group (non-pianists). First, the study avoided including people in this group who had some musical ability, either with the piano or another instrument. Second, as revealed in the previous literature, some factors can increase the performance in using BCI management systems, such as the practice of tasks that involve digital motor coordination (video games, typing, other professions that require precision motor skills, etc.) and the practice of sports that result in a substantial improvement in motor coordination. Therefore, an attempt was made to choose participants in such a way as to minimize these aspects. Table 2 summarizes the characteristics of the control group.

The non-pianists in the control group were asked about their musical knowledge and musical practice. Those who volunteered for the control group and showed some musical knowledge and/or practiced with musical instruments of any kind (piano or other) were not selected. Likewise, those who practiced a sport at an almost professional level were not selected. Furthermore, all members of the control group had a normal level of digital activity that did not go beyond one or two hours a day spent on work-related typing on a computer keyboard and all practiced sport only sporadically.

The time that the participants in the control group spent on motor practice is contrasted with that of the pianists, as these all began their piano studies at around 10 years of age and had had professional careers lasting between 10 and 15 years. Their beginning at an early age and their years of practice was estimated to translate to about 6 h of daily finger practice (more on some days). This amount undoubtedly exceeded that of the control group, not only in terms of motor coordination but also long-term musical orientation, which has been shown to create characteristic brain structures. In the case of the pianist group, the hours spent on other fine motor coordination practices were also collected, but these values were very close to those of the control group and were also irrelevant compared to the hours of musical practice.

### 3.2. Resources Used and EEG Acquisition

**Figure 1:**
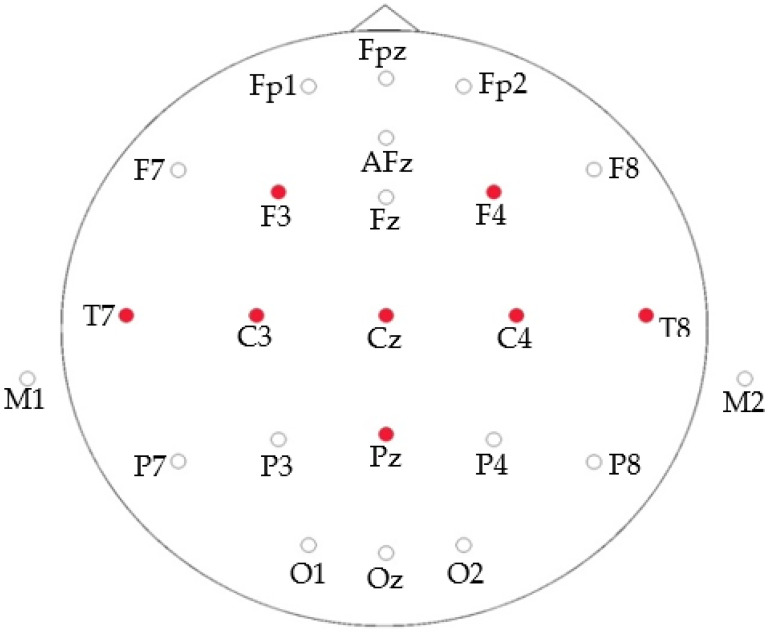
Electrodes location.

Enobio-8 is the wireless and portable sensor system that we have used for EEG recording. This device consists of a neoprene helmet with 39 holes to cover the main positions of the distribution according to the 10–20 system. The helmet makes it possible to use dry and wet electrodes. The electrodes chosen for this experiment are of the dry type. Eight electrodes are used in this case located at F3, F4, T7, C3, CZ, C4, T8 and Pz according to 10/20 system. Figure 1 shows the location of the electrodes.

We must also take into account two more electrodes which will be used as grounding for the system, which allows the rest of the signals to be correctly referenced. They are attached to an Ag/AgCl EEG measurement patch with a conductive semi-liquid gel with low impedance, placed under the lobe of each ear. The electrical signals from the cortex are collected and subsequently sent to a computer via Bluetooth.

The accuracy of the system is approximately 0.005 µV. Knowing that the voltage we are working with ranges between 10 and 100 µV, the accuracy of the results is at least 0.005%.

The channels were recorded with a sampling frequency of 500 Hz. The recordings had a dynamic range of ±100 μV for all sessions. A notch filter at 50 Hz was enabled. We placed two electrodes at the mastoid bone as an EEG ground.

In addition, different standard software is used in the experiment for signal acquisition and post-processing:

- OpenVibe 2.2.0. This program is used as an interface between the computer and the user since it shows mental tasks and produces feedback. OpenVibe can also be used as a generic real-time EEG acquisition, processing and display system, allowing the online analysis of results, applying a feature extractor and classifier throughout the course of the experiment to perform the feedback task.
- Matlab R2016b. MATLAB, through toolboxes dedicated to Machine Learning, makes it possible to carry out an offline analysis, which will allow us to test the characteristics extractor and the classifier with different time windows.

### 3.3. Experimental Timeline

In order to find out if the research hypothesis has been fulfilled, the experiment was carried out in which the EEG signal of a group of pianists and a control group was analyzed. To do so, the motor imagery of two dichotomous movements was used, one of the left hand and the other of the right hand, which served to control a BCI system, and the success rate of each individual and each group was analyzed.

The most widely used training method for the BCI (and, therefore, followed in this study) is that based on the Graz Principle [42], developed at the Institute of Neural Engineering of the Technological University of Graz (Austria). This procedure is divided into two stages:

- Stage 1. Training the system to recognize the signal, which allows the computer to record brain information, which will be used to extract some spatial characteristics and divide them into classes. With this training, feature extraction is adjusted, and the classifier algorithm is trained.
- Stage 2. User feedback to teach subjects how to control the BCI. At this stage, the subject receives feedback from the computer and can see how the system perceived their actions, analyzing and modulating their own brain activity. This feedback consists of a blue bar shown in the screen that matches the interpretation of the Machine Learning algorithms trained in the first stage.

Three sessions of three trials each with 40 sequences per trial were carried out with each subject. The first trial of each session corresponds to the first stage described above, followed by the second trial, which develops the second stage. The third trial repeats the second stage in order to increase the number of data collected. Table 3 summarizes the above.

### 3.4. EEG Processing

In the first trial, no feedback was produced, since only data collection was carried out, which will be used to train the system through a feature extraction and a classifying algorithm. To do this, a spatial filter obtained by Common Spatial Pattern (CSP) and a Linear Discriminant Analysis (LDA) classifier were used, as will be explained later. However, for trials 2 and 3 of each session, a feedback was performed that consists of showing the user what the BCI system interprets they are thinking, according to the previously trained algorithms (online analysis). However, once the data collection of each subject was completed during the three sessions, an offline analysis was carried out, using as well CSP and LDA.

CSP is a mathematical technique used in signal processing to separate multivariable signals into sub-components with different variances. The CSP method was first proposed under the name Fukunaga-Koontz Transform in [43] as an extension of Principal Component Analysis (PCA) and has been widely used in BCI to maximize the distance between two classes of motions.

A CSP filter maximizes the variance of filtered EEG signals from one motion class while minimizing it for signals from the other class. The development of this technique comes naturally when we try to maximize the difference of variances between the two signals by spatially filtering them.

LDA is a Machine Learning technique used to perform supervised linear classification, based on a range of observations that can be divided into groups or classes [44]. The problem is basically to assign the right class to each observation. Linear classifiers are those that base their decision according to some hyper plane by assigning each class to one side of the subspace evaluated.

LDA is a simple and very stable technique, which will allow us to perform the type of classification we need without the use of any other parameter. This method is based on the assumption that we have two classes that follow a normal distribution. For each class, the parameters of mean and variance are modeled to get the distribution that best describes it and then the Bayes’ theorem is used to calculate the probability of belonging to each of the classes.

The combination of CSP and LDA has been widely used in MI-based BCI to maximize the distance between two classes of movements [9,45]. There are some methods which observe the different activity between bilateral sides of hemispheres during the imagery, but some of these methods are too complex or demand too much computing time, so it is hard to apply them in real-time applications. CSP and LDA have also the characteristic of being 2-class methods. A CSP filter maximizes the variance of filtered EEG signals from one motion class while minimizing it for signals from the other class. After using this method, the goal is to train a classifier in such a way that it is able to estimate the class to which an observation belongs from observations of which we know the class, so LDA was used.

### 3.5. Experiment Deployment

The approximate time necessary for setting up and conducting the experiment was 45 min for each session, which required a certain availability of the subjects. Between each session, it was ensured that there was a rest period for each subject of at least four days. Between trials we provided 10 min in order to let the volunteer rest.

The procedure is similar as that depicted in BCI Competition IV [46]. The subjects were right-handed and had normal vision. All volunteers were sitting in a chair, with a flat screen monitor placed 1 m away at eye level.

Taking the existing literature into account, the subjects were asked to imagine specific movements of the hands and fingers. This movement will consist of a drumming of the fingers of the hand, accompanied by a swinging of the wrist up and down. Before carrying out the experiment, each subject was recommended to practice it in a real way to assimilate the physical sensation and, subsequently, to repeat it in an imagined way. Furthermore, during the experiment, it was recommended that subjects be careful not to make any parasitic movements with their hands, eyes or head, which could have jeopardized the accuracy of the calculation. This fact was explained at the beginning of the experiment and it was discussed in the following sessions.

**Figure 2:**
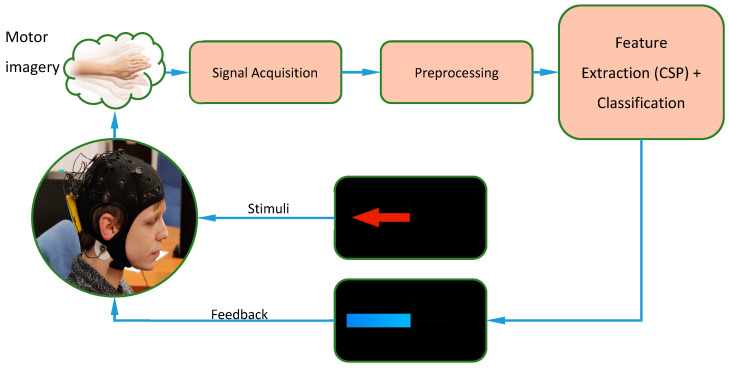
Experiment deployment.

The structure of the entire experiment can also be described with the diagram shown in Figure 2. Each session would begin with the donning of the helmet for EEG analysis. Subsequently, a first phase would be carried out with each subject that will allow adjustments to both the extraction of characteristics and the training of the classifier (online analysis). Once the parameters are adjusted, the system is ready to be able to present real-time feedback on the screen. Subsequently, two iterations are carried out in which the subject can see in real time the interpretation that the system makes of his movement imagery. This process will be repeated over three days and the data will later be analyzed offline.

**Figure 3:**
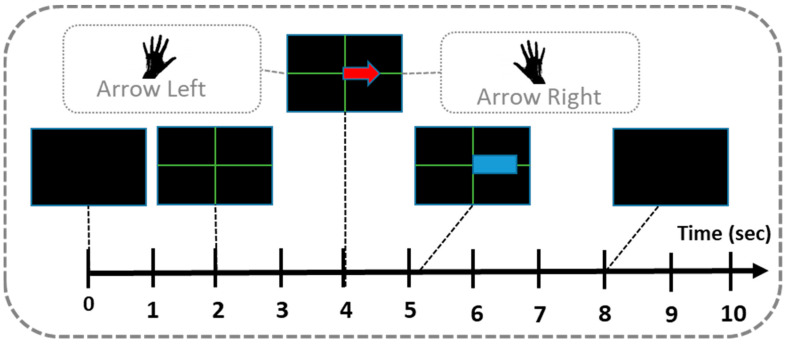
Timing of the brain-computer interface (BCI) System.

A timing detail of each sequence can be seen in Figure 3. In second 0, the system presents a back screen. In second two presents a green cross and, 2 s later appear and arrow for 1.25 s. The arrow indicates de MI that the user must done. If the arrow point at left, the user must imagine the movement of the right hand and, if the arrow point left the user must imagine the movement with the other hand. Only in Trials 2 and 3 the system shows feedback as a blue line at second 5 and, it changes its longitude according to the output of the classifier. At second 8 the screen turns black and below there is a random time near 2 s in order to avoid synchronization between user and the timing protocol. For the presentation of the stimuli we used the previously named OpenVibe software.

### 3.6. Offline Analysis

Once the data is recorded and the experimental phase is ended, we repeated the computation of the EEG offline but this time with a much more elaborate processing, since during the offline analysis the subject is not present, and then a more time-consuming algorithm can be used.

MATLAB is used to preprocess the subjects’ EEG data. In the offline analysis, we enabled a notch filter at 50 Hz. This is due to the AC lines of the electrical supplies, which can introduce oscillations at 50 Hz and hence, noise oscillations in the EEG recordings. In addition, the data were low-pass filtered and high-pass filtered with cut-off frequencies of 1 Hz and 100 Hz, respectively, in order to eliminate frequencies which are impossible to be produced by the brain, thereby improving the signal cleanliness. Detection of artifacts is carried out with visually routines. An “artifact” can be described as any component of the EEG signal that is not directly produced by human brain activity, but induced by muscle activity, cardiac activity, respiration, and mainly blinks. The proximity of the eyes to frontal electrodes and the intensity of the blinking can produce a big distortion of the EEG that is sometimes impossible to be cleaned. To the trained eye, it is easy to detect, in an EEG graph, the presence of artifacts and their importance. The most notable artifacts are removed by rejecting the piece of data containing the artifact, forcing to discard in some cases the whole data of some volunteers.

To obtain the performance results of each subject, a combination of the CSP and LDA algorithms executed with leave-one-out cross validation has been used. Leave-one-out cross-validation is a certain cross-validation case where the number of folds is equal to the instances in the data set. Hence, the learning algorithm is applied one time for each instance, using all the other measurements as the training set and using the selected instance as a unique item test-set.

### 3.7. Statistical Analysis

In order to appreciate differences in the performances between groups based on statistical support, some verification must be done. Firstly, a Shapiro-Wilk test has been performed with the goal of checking normal distribution. In this test, the null hypothesis (H0) considers that the data set came from a normally distributed population. The Shapiro-Wilk Test is very appropriate for small sample sizes (<50 samples).

Next, the parametric Welch’s T-test has been used to estimate if pianists’ performances differ significantly from non-pianists’ achievements. This is a two-sample test which is used to test the hypothesis that two populations have equal means. So, the null hypothesis (H0) considers equal means between the two groups under discussion.

In both cases, a significance level α = 0.05 is assumed to be appropriate.

## 4. Results

To carry out the experiment explained above, samples were taken from 8 subjects, 4 of whom were pianists and the other four were not (non-musicians), functioning as a control group.

In a controlled motor imagery BCI experiment, accuracy of between 80% and 90% is expected after 6–9 training sessions of 20 min each [47]. Different investigations have presented different thresholds for the “efficiency” of the BCI, but a reference value for acceptable results is 70% [29]. However, according to the state of the art, certain subjects may have difficulties using BCI systems.

Control of BCI systems requires learning both the system and the user; there must be mutual adaptation. Due to this, it is expected that the yields in the classification will increase with the user’s training. Thus, the first two were introductory and learning, achieving the best performance on the third and last day.

These results are obtained from the offline analysis with the MATLAB program, using the 80 sequences together from trials 2 and 3 combined, from each session. Due to this, we will focus on the presentation of the results concerning the last day (session 3). The first trial of each day was used to train the CSP+LDA used in the online analysis in order to provide the closed loop feedback to the user.

**Figure 4:**
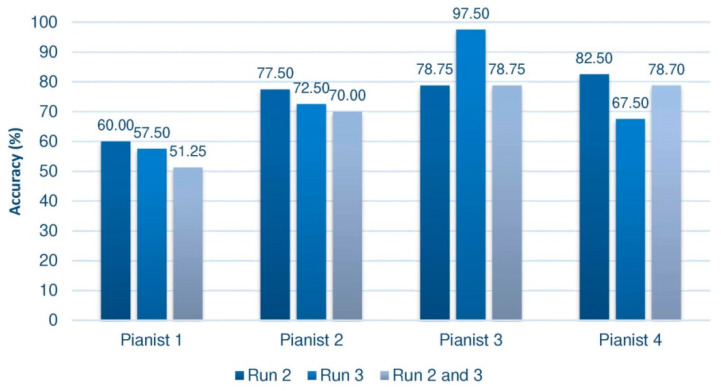
Offline results of the group of pianists in the third session. Measurements in Run 2 and 3 were taken from feedback trials.

**Figure 5:**
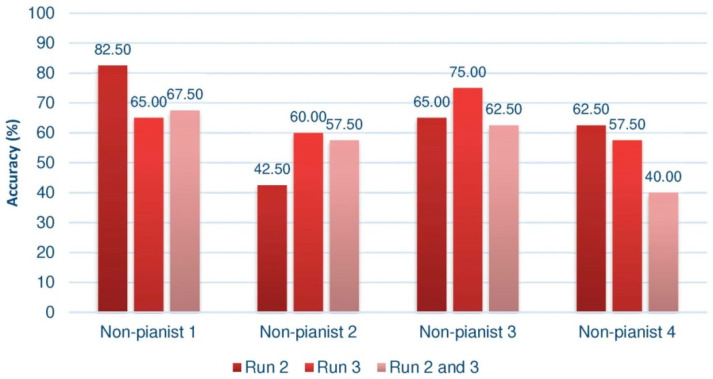
Offline results of the non-pianist group in the third session. Measurements in Run 2 and 3 were taken from feedback trials.

Offline processing allows various results to be obtained, since the cross-validation technique can be applied to different subsets of data. This analysis will be done on the second and third trials of the third session, in which the user had feedback. Various authors such as [48] consider the first trial to be a training phase to give participants the opportunity to learn how to carry out the motor imagery, concentrating their analysis on the following phases. Moreover, the performance on the set of the 40 sequences of the second trial will be studied and we will also proceed with the 40 sequences of the third trial. In addition, independently, the 80 sequences of both tests will be taken together. The results corresponding to the group of pianists are summarized in Figure 4, and those of the control group, below (Figure 5).

**Figure 6:**
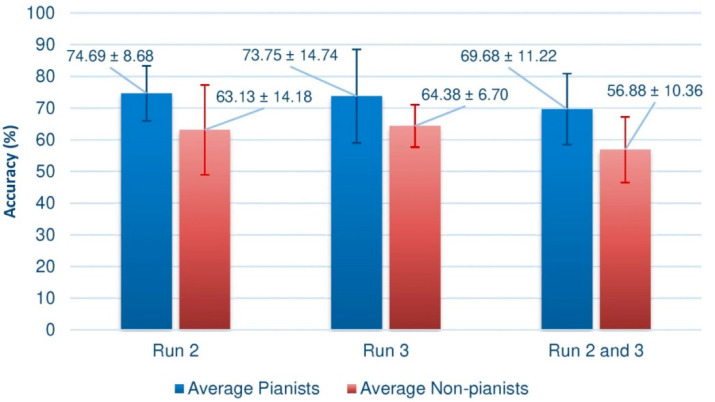
Comparison of offline results in both groups. Measurements in Run 2 and 3 were taken from feedback trials.

As stated above, a Shapiro–Wilk test can determine whether the data present a normal distribution. The results indicated that the data was normally distributed (*p*-values > 0.05). To compare the means of both groups, we performed the parametric Welch’s T-test. The results indicated in an overall consideration that the performance of the pianists differed significantly from that of the non-pianists (*p*-value = 0.0344445, α = 0.05). Figure 6 indicates the comparison between Runs (2, 3 and 2-and-3), showing that, in all circumstances, the difference between the averages is large enough to be statistically relevant and below the significance level of 0.05.

**Figure 7:**
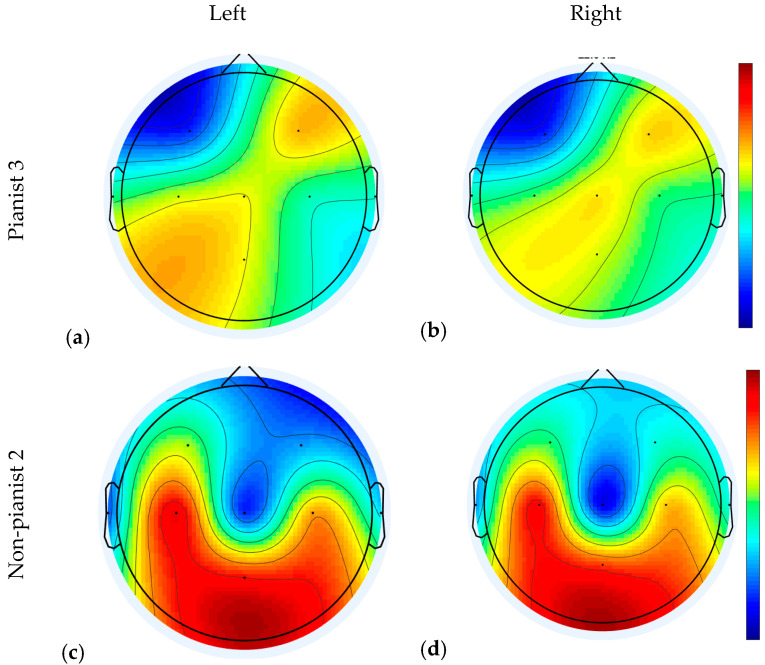
Examples of Projected electroencephalogram (EEG) signal after a Common Spatial Pattern (CSP) filter for Left and Right MI of Pianist 3 and Non-Pianist 2. Subfigures (**a**) and (**b**) belong to Pianist 3 (left/right, respectively), and subfigures (**c**) and (**d**) belong to Non-pianist 2 (left/right, respectively).

The scalp topography illustrates how the physiological sources project to the scalp. Figure 7 shows two examples of projected EEG signal after a CSP filter [49]. Subplots (a) and (b) present topographies of Pianist 3 in right and left MI along Run 2 and 3 at 22 Hz. Subplots (c) and (d) present topographies of Non pianist 2. All of them were computed along Run 2 and 3 at 22 Hz. Dense red or blue areas show where the greatest differences in the projected signals were found. As can be seen, for Pianist 3, channel F4 and Cz were the most actives, being C3 and C4 in the medium scale of colors. However, in Non-Pianist 2, channels C3, C4 and Pz are marked with strong red color. In general, we observe a greater area of activation in the Non-Pianist subject as compared with that in the Pianist participant.

## 5. Discussion

Despite the disparity of results depending on the set of sequences analyzed, some conclusions can be outlined. Paying attention to the group of pianists, we can see that almost all of them, whatever the test taken, achieve yields in the third session greater than 70%. Pianist 1 would be a possible BCI-Inefficient user, although, as we have seen, we could only confirm this after 6 or 9 training sessions. This kind of users would require a different approach in order to improve their performance. In this sense, some studies have been developed with the aim of deploy a specific design of the experiment [50]. This would explain why the subject has not evolved or shown better performance. Among all of them, Pianist 3 stands out, reaching an almost perfect efficiency of 97.5% in the third trial. This result is very striking, considering that these rates are usually reached in more advanced sessions.

A common aspect in both groups is the drop in performance in the third trial, which occurs in 5 of the 8 cases. Mood, motivation, frustration, etc., are factors that determine the performance of the BCI. In total, the trial lasts an hour, and it is quite difficult to maintain the optimal concentration state during that time, carrying out the movement imagery tasks. Tejedor [51] calls it the “maturation effect”, so that the optimum point of the results of a group of participants who are undergoing an experiment would be between the beginning (where the procedure is not yet mastered) and the end (where tiredness, fatigue, lack of interest, etc. come into play). In addition to this concept, Tejedor indicates that we have also to bear in mind the “experimental mortality”, this is, the dropout of the volunteers along the experiment due to a lack of interest or change in the familiar/labor circumstances. Frustration can also trim the learning curve when we are considering inexperienced subjects. For such reason, motivation is an important issue to take into account in order to get acceptable results [52].

Comparing both figures, it is quite evident to conclude that the performance of pianists is, in general, higher than that of the control group, which seems to suggest that the proposed research hypothesis is fulfilled. Indeed, when calculating the mean of the results in each of the trials of the third session (the second, the third, and the set of second and third), we observe that the average is always higher in the pianists, as shown by the Figure 6, where the standard deviations of each mean value are also indicated.

Not only is it significant that the means are superior, regardless of the way in which the data is processed, but also that the performance is always close to 70%. Furthermore, recall that these results are impoverished by the presence of a supposed subject with BCI illiteracy, which would also explain why the mean of the set of trials 2 and 3 remains a few hundredths away from the optimal value of BCI performance. In the case of the group of non-pianists, except for some partial results, this figure is not reached. It is striking that the fourth subject of the group of non-pianists has an extremely low result when joining the two sessions.

The present study was intended to examine the analysis of the performance of a BCI device by means of the motor imagery carried out by pianists. In this sense, this work has synthesized some relevant results regarding the neurobiological differences that musical practice entails, showing that said functional specialization entails significant anatomical differences. Moreover, the foundations of the technology of the brain-computer interfaces have been exposed. These artifacts are able to detect the electrical activity of the brain with electrodes that perform an EEG and, through processing with machine learning algorithms, this biological signal is transferred into an order that can have different applications, such as the control of smart homes or robots.

The musician’s brain is a paradigmatic case of neuroscience. However, in the existing literature, no previous study has been found that analyzes the performance of musicians with BCI systems, using motor imagery. The literature does seem to show that having musical prowess suggests better performance, but in the absence of a specific study in this regard, it was questionable whether, in fact, musical training and structural differences in the brain of musicians entailed better performance. To answer this hypothesis, an experimental procedure based on BCI has been designed for this paper.

Music is a global activity that involves the development of different capacities and intelligences: it improves memory, concentration, reading, self-esteem, emotional intelligence, psychomotricity, etc. In this sense, given the need to control BCI devices in the future, we can affirm that musical practice could improve motor coordination as well as neural plasticity, as revealed in the literature review, hence favoring their optimal use. To conclude that musical training is one of the factors that favors performance with motor imagery does nothing more than claim the importance of musical education in our society.

## 6. Conclusions

In this work, the performance of a set of pianists as they interacted with an MI-based BCI system was assessed and compared with a control group. The Common Spatial Patterns (CSP) and Linear Discriminant Analysis (LDA) machine learning algorithms were applied to the EEG signals for feature extraction and classification, respectively. The results revealed that the pianists achieved a higher mean level of BCI control—by means of MI—during the final trial (74.69%) in comparison to the control group (63.13%).

Regarding the above, it can be concluded that there seems to be indications that musical training is indeed a factor that improves the performance of a BCI device through movement imagery. As mentioned before, the performances achieved by the pianists in the last trial are on the order of 10 points higher than the non-pianists, regardless of the data set analyzed, which suggests that the previous hypothesis is true.

In future research, these results could be completed, on the one hand, by expanding the sample and, on the other, by supporting training for several sessions in order to reach more definitive conclusions. With this, we could explore the improvement of the performance of the two cases we found that were BCI inefficient. As we cannot venture a hypothesis based on only three sessions, with a greater number of measurements, we would be able to explore the evolution of these participants and deepen our understanding of so-called BCI illiteracy.

## Figures and Tables

**Table 1:** Description of the group of pianists.

Characteristic	Values	
Subjects	4	
Sex	2 men and 2 women	
Level of education	2 students and 2 professionals	
	**Mean**	**Standard Deviation**
Age (years)	24.50	±1.50
Time playing (years)	12.75	±1.78
Musical practice (hours/day)	5.75	±1.47

**Table 2:** Description of the control group.

Characteristic	Values	
Subjects	4	
Sex	2 men and 2 women	
	**Mean**	**Standard Deviation**
Age (years)	32.75	±5.44
Sport (hours/week)	3.50	±1.11
Digital practice (hours/day)	1.63	±0.96

**Table 3:** Classic training method.

	Trial 1	Trial 2	Trial 3
**Session 1**	Training	Feedback	Feedback
	40 sequences	40 sequences	40 sequences
**Session 2**	Training	Feedback	Feedback
	40 sequences	40 sequences	40 sequences
**Session 3**	Training	Feedback	Feedback
	40 sequences	40 sequences	40 sequences
